# The International Network for Evaluating Outcomes of very low birth weight, very preterm neonates (iNeo): a protocol for collaborative comparisons of international health services for quality improvement in neonatal care

**DOI:** 10.1186/1471-2431-14-110

**Published:** 2014-04-23

**Authors:** Prakesh S Shah, Shoo K Lee, Kei Lui, Gunnar Sjörs, Rintaro Mori, Brian Reichman, Stellan Håkansson, Laura San Feliciano, Neena Modi, Mark Adams, Brian Darlow, Masanori Fujimura, Satoshi Kusuda, Ross Haslam, Lucia Mirea

**Affiliations:** 1 Canadian Neonatal Network, Maternal-Infant Care Research Centre, Mount Sinai Hospital, 700 University Avenue, Toronto, Ontario M5G 1X6, Canada; 2 Australia and New Zealand Neonatal Network, Royal Hospital for Women, Level 2, McNevin Dickson Building, Sydney Children’s Hospital, Randwick, NSW 2031, Australia; 3 Swedish Neonatal Quality Register, Department of Women’s and Children’s Health, Uppsala University, 751 85 Uppsala, Sweden; 4 Neonatal Research Network Japan, Department of Health Policy, National Center for Child Health and Development, 2-10-1 Okura, Setagaya-ku, Tokyo 157-8535, Japan; 5 Israeli Neonatal Network, Gertner Institute for Epidemiology and Health Policy Research, Sheba Medical Centre, Tel Hashomer 52621, Israel; 6 Swedish Neonatal Quality Register, Department of Pediatrics, Umea University Hospital, SE-901 85 Umeå, Sweden; 7 Spanish Neonatal Network, Unidad Neonatal Barakaldo, Plaza de cruces s/n, 5ª Planta, Unidad Neonatal, Barakaldo 48903, (Bizkaia), Spain; 8 UK Neonatal Collaborative, Imperial College London, Chelsea and Westminster Hospital Campus, London SW10 9NH, UK; 9 Swiss Neonatal Network, Division of Neonatology, University Hospital Zurich, Frauenklinikstrasse 10, CH-8091 Zürich, Switzerland; 10 Australia and New Zealand Neonatal Network, University of Otago, Christchurch, 2 Riccarton Avenue, PO Box 4345, Christchurch 8140, New Zealand; 11 Neonatal Research Network Japan, Osaka Medical Center and Research Institute for Maternal and Child Health, 840 Murodo-cho, Izumi, Osaka 594-1101, Japan; 12 Neonatal Research Network Japan, Maternal and Perinatal Center, Tokyo Women’s Medical University, 8-1 Kawadacho, Shinjuku-ku, Tokyo 162-8666, Japan; 13 Australia and New Zealand Neonatal Network, Women’s and Children’s Hospital, Adelaide, Level 2, McNevin Dickson Building, Sydney Children’s Hospital, Randwick, NSW 2031, Australia

**Keywords:** Very preterm infants, Very low birth weight infants, Neonatal intensive care unit, Neonatal networks, Comparative analysis, Neonates, Quality improvement

## Abstract

**Background:**

The International Network for Evaluating Outcomes in Neonates (iNeo) is a collaboration of population-based national neonatal networks including Australia and New Zealand, Canada, Israel, Japan, Spain, Sweden, Switzerland, and the UK. The aim of iNeo is to provide a platform for comparative evaluation of outcomes of very preterm and very low birth weight neonates at the national, site, and individual level to generate evidence for improvement of outcomes in these infants.

**Methods/design:**

Individual-level data from each iNeo network will be used for comparative analysis of neonatal outcomes between networks. Variations in outcomes will be identified and disseminated to generate hypotheses regarding factors impacting outcome variation. Detailed information on physical and environmental factors, human and resource factors, and processes of care will be collected from network sites, and tested for association with neonatal outcomes. Subsequently, changes in identified practices that may influence the variations in outcomes will be implemented and evaluated using quality improvement methods.

**Discussion:**

The evidence obtained using the iNeo platform will enable clinical teams from member networks to identify, implement, and evaluate practice and service provision changes aimed at improving the care and outcomes of very low birth weight and very preterm infants within their respective countries. The knowledge generated will be available worldwide with a likely global impact.

## Background

The global incidence of preterm birth is on the rise [1]. In Canada the incidence of preterm birth (<37 weeks gestational age) has increased from 6.3% in 1981 to 7.7% in 2009 [2,3]. Although infants born at a very low birth weight (VLBW, <1500 g) and/or very preterm (VPT, <32 weeks gestational age) make up only 14% of all preterm births in Canada [3], they are of significant public health importance due to their high risk of mortality and childhood morbidities. These morbidities include developmental problems, cerebral palsy, cognitive delay, blindness, and deafness [4,5], with an estimated lifetime cost of CAD$676,800 per preterm infant with permanent disability [6]. Therefore, it is important to identify strategies that will reduce the risk of adverse outcomes suffered by VLBW and VPT infants and improve quality of life for these infants.

Various national neonatal networks, such as the Australia-New Zealand Neonatal Network (ANZNN) [7], Canadian Neonatal Network (CNN) [8], Israeli Neonatal Network (INN) [9], Neonatal Research Network of Japan (NRNJ) [10], Swedish Neonatal Quality Register (SNQ) [11], and UK Neonatal Collaborative (UKNC) [12], have been established to collect data from their constituents and identify trends in the outcomes of VLBW infants and benchmark the performance of their respective centers. Although advances in neonatal care between the 1960s and the 1990s resulted in significant reductions in mortality and morbidity for neonates [13-16], recently some networks, including the CNN, have observed a halt in progress or even worsening of outcomes [13,17-19].

Even for those neonatal networks where continued improvements in outcomes have been reported, there remains significant variation within and between networks. For example, several comparative studies have identified differences in mortality rates in neonates from separate networks, regions, or countries [20-27]. In one such study, Draper et al. reported that among 10 European regions, the overall survival rate for VPT infants varied from 74.8% to 93.2% [21]. More recently, in 2012 population data from the UKNC showed a greater than three-fold variation between regional networks in the percentage (range 4.7% to 16.6%) of infants born at <30 weeks gestation and admitted to neonatal units who died at ≤28 days of age [27]. Comparison of selected Australian and Scottish neonatal intensive care units (NICUs) detected a lower risk-adjusted mortality rate for VPT/VLBW infants in Australia compared with Scotland [28].

However, studies of a single or small group of sites are subject to selection bias, which can lead to erroneous conclusions when the results are generalized to the larger population. Furthermore, comparisons of mortality alone may be misleading as mortality may be declining at the cost of increasing morbidities. Measurement of mortality as an indicator of care is also a contentious issue as there are marked variations in practice between countries including initiation or withholding of resuscitation at earlier gestational ages [29]. Thus, this protocol for the International Network for Evaluating Outcomes of Neonates (iNeo) was developed to examine neonatal morbidities in conjunction with mortality using population-based data, and assess variations in practice that impact outcomes between and within countries.

### Rationale

Over the past 5 years, collaborations have been initiated between the CNN, NRNJ, ANZNN, and SNQ. The first ever population-based retrospective comparison between countries showed that a composite outcome of mortality or any major morbidity (bronchopulmonary dysplasia [BPD], severe neurological injury, ≥stage 3 retinopathy of prematurity [ROP], nosocomial infection [NI], and ≥ stage 2 necrotizing enterocolitis [NEC]) was lower in VLBW infants in Japan compared with Canada. In-depth analyses revealed higher rates of severe neurological injury, NEC, and NI among NICUs in the CNN, whereas rates of BPD and ROP were higher in NRNJ NICUs [30]. Comparisons between the CNN and the ANZNN for VPT infants identified that while there was no difference in mortality, the ANZNN had significantly lower rates of severe neurological injuries, ROP, NEC, and BPD, but higher rates of early onset sepsis and air leaks and longer mean length of stay [31,32]. Our latest comparisons indicated that rates of adverse outcomes at each gestational age were lower in Sweden compared with Canada (unpublished data).

Differences in the outcomes of VLBW and VPT infants between Canada and other countries could be due to any number of factors including differences in population characteristics, severity of illness, processes of care, or delivery of health care. Informal discussion has confirmed wide variations in these factors between networks. For example, compared with Canada, the use of non-invasive respiratory support is higher in Europe, the use of breast milk is higher in Japan and Scandinavia, and the use of echocardiography by neonatologists for hemodynamic monitoring is routine in Japan. Differences in the type of intervention and process of administration may underlie at least some of the variations in outcomes. In addition, there are extreme variations in health services delivery and receipt. For example, the number of outborn, very preterm infants is significantly lower in the ANZNN compared with the CNN [32]; the use of respiratory therapists is practically non-existent in European countries, whereas they play a prominent role in North American institutions; and shift work is more prevalent among junior doctors in Europe and Australia [33] compared with Canada.

Given the variation in mortality and morbidity between countries, it is important to first characterize factors underlying these differences, and then identify areas and approaches to improve neonatal care specific to each network. Care provision to VLBW and VPT infants is a highly selective health service where specialized units deliver the majority of such care (approximately 80% of VLBW and VPT infants are admitted to tertiary NICUs), and consumes extensive resources, both in terms of the per-diem cost of caring for such a neonate in the NICU and cumulative lifetime costs. To improve outcomes and reduce health care costs globally, we need to embrace the concept of collaborative sharing and learning, assess the variation in practices between countries/networks, identify evidence-based practices associated with improved outcomes, and apply these practices to deliver optimal health care to fragile neonates.

Currently, informal and indirect comparisons can be made from the reports published by each national network. However, criticisms of such indirect or post-hoc comparisons include lack of adjustment for differences in baseline infant and maternal characteristics, differences in definitions of outcomes and their measurement, and variations in physical, environmental, and human factors (e.g. training system and associated working conditions of physicians on duty day and night, differences in nursing care and nurse:beds ratio, differences in regionalization system, and the rate of maternal transfer for extremely preterm fetuses). A system of data standardization and an understanding of the context for comparison are urgently needed to enable valid comparisons between networks. This can only be achieved through an international collaboration where the knowledge users and decision makers are involved from the start of the process and continuously through to knowledge translation. Analyses of network-level data using all eligible infants will provide a more accurate estimate of the effectiveness of an intervention in a pragmatic setting, rather than just a measure of efficacy proven in a controlled study setting.

### Network objectives

The specific aims of iNeo are to:

– 1. Compare outcomes for infants born with VLBW (weighing <1500 g) and VPT (<32 weeks gestation) among eight national neonatal networks spanning nine countries.

– 2. Identify site-level physical, human, and environmental characteristics, as well as care practices that are associated with variations in outcomes.

– 3. Identify clinical and organizational practice improvements relevant to each network.

– 4. Implement and continually evaluate the impact of evidence-based clinical and organizational practice changes in NICUs within the iNeo networks.

The establishment of the iNeo collaboration will enable the following: i) collection and integration of individual-level data from population-based networks on outcomes, characteristics, practices, and culture of the member sites; ii) evaluation of the impact of practice and outcome variations to identify the best models of health service delivery (incorporating medical and other extraneous factors); iii) feedback to units of their standing in reference to each and all other networks; iv) empowerment of units to embrace implementation of evidence-based practice changes for quality improvement; and v) performance of ongoing cycles of translating knowledge-to-action through continuous auditing. Ultimately, this will improve outcomes for VLBW infants across the iNeo member networks.

## Methods/design

### Overview

The comparison of neonatal mortality and morbidity between the eight member networks will be conducted using four years of retrospective data collected between January, 2007 and December, 2010. Subsequently, a strategy will be designed to collect additional data and assess differences in physical, environmental, and human characteristics, and care practices associated with variations in outcomes between networks. Once identified, clinical and organizational practice improvements will be implemented within networks using the Evidence-based Practice for Quality Improvement (EPIQ) method [34,35]. The effect of practice change implementation will be measured using ongoing data collection within each network. The total study period will be five years (January 2013 to December 2017). Comparison between the networks will be completed by early 2014, associations between external factors/care practices and outcomes identified by the end of 2014, and selected practice changes implemented by mid 2015. This will be followed by a two and a half-year period of continuous quality improvement within the networks.

### Participating networks

The following neonatal networks have agreed to participate in the iNeo project: Australia-New Zealand Neonatal Network (ANZNN), Canadian Neonatal Network (CNN), Israeli Neonatal Network (INN), Neonatal Research Network of Japan (NRNJ), Spanish Neonatal Network (SEN1500), Swedish Neonatal Quality Register (SNQ), Swiss Neonatal Network (SNN), and UK Neonatal Collaborative (UKNC) (see Table 1). Overall, this project will be collecting data from a total of 251 NICUs in nine countries caring for approximately 23,000 to 24,000 VLBW neonates per year. All the participating networks have a common mandate to collect, analyze, and benchmark performance and outcomes of their respective NICUs. We have carefully avoided networks that only include highly specialized units in order to obtain robust population-based estimates. All participating networks have confirmed the feasibility of data collection from >75% of all VLBW and VPT infants born within their country. The approximately 25% of infants missing from some of the networks are those considered to be at the higher end of maturity (>1300 g birth weight or >30 weeks gestation) who do not require intensive care support. These infants are relatively stable and do not represent a significant burden to NICUs or health care services in general.

**Table 1:** Characteristics of networks participating in the International Network for Evaluating Outcomes of Neonates (iNeo)

**Network**	**Australia and New Zealand Neonatal Network**	**Canadian Neonatal Network**	**Israeli Neonatal Network**	**Neonatal Research Network Japan**	**Spanish Neonatal Network**	**Swedish Neonatal Quality Register**	**Swiss Neonatal Network & Follow-Up Group**	**UK Neonatal Collaborative**
Country	Australia and New Zealand	Canada	Israel	Japan	Spain	Sweden	Switzerland	UK (England)
Level III NICUs in the country	23 + 6	30	23	93	n/a	7	9	45
Level III NICUs in the network	29	30	23	73	36	7	9	44
Number of inhabitants	Australia: 23 million NZ: 4.4 million	34 million	7.9 million	126 million	47 million	9.5 million	8 million	52 million
Number of births/year	Australia: 300,000 NZ: 60,000	380,863	166,000	1,071,304	497,023	110,000	80,000	687,000
Number of eligible NICU admissions/year (<32 wks gestation/<1500 g)	3,500	2,700	1,500	3,700	2,600	900	800	7,700

### Database variables

A detailed review of all the data items collected by each of the participating networks has been conducted and the elements common to all networks (e.g. gestational age, birth weight, sex, etc.) included in a minimum dataset (see Additional file 1 for full list of data variables). Data items that are collected by all networks in slightly different formats (e.g., nosocomial infection, which can be defined by using a cut-off of 2 days, 3 days, or 7 days) have been standardized across all the networks by consensus of the network directors. Some networks already extract data from their databases according to the iNeo definitions, while others have agreed to redefine their original data formats as an ongoing process to ensure consistency and facilitate comparisons over time. The variable definitions have been mapped to the ICD-10 [36] and SNOMED [37] dictionaries.

### Ethics, data collection, and dissemination

All participating networks have obtained ethics/regulatory approval or the equivalent from their local granting agencies to allow for de-identified data to be sent to the iNeo Coordinating Centre at the Maternal-Infant Care Research Centre, Mount Sinai Hospital, Toronto, Canada. The Coordinating Centre has been granted Research Ethics Board approval for the development, compilation, and hosting of the iNeo dataset, and all networks have signed data transfer agreements with the iNeo Coordinating Centre. Privacy and confidentiality of patient and unit-related data will be of prime importance to the iNeo collaboration, and data collection, handling, and transfer will be performed in accordance with the Canadian Privacy Commissioner’s guidelines, the Personal Information Protection and Electronic Documents Act, and any other local rules and regulations. No data identifiable at the patient level will be collected or transmitted, and only aggregate data will be reported. For all stages of the project, participating units will be assigned a code by their own network prior to data transfer into the iNeo dataset so that units remain anonymous within the iNeo collaborative. Following data analysis, findings will be disseminated within networks by their own network coordination team and not by the iNeo central team.

Following completion of the study in 2017, the data will be kept at the iNeo Coordinating Centre for a further two years before being returned to the originating networks unless otherwise agreed by the member networks.

### Comparisons of neonatal outcomes between networks

#### Outcomes

The primary outcome for comparison between the networks will be a composite indicator of mortality or any of the four major neonatal morbidities (severe neurological injury, severe ROP, NEC, and BPD). Mortality will be defined as death due to any cause prior to discharge home. Severe neurological injury will be defined as ≥ stage 3 intraventricular hemorrhage (IVH) with ventricular dilatation according to the criteria of Papile et al. [38], or parenchymal injury (including periventricular leukomalacia) with or without IVH. Severe ROP will be defined as ≥ stage 3 according to the International Classification [39], or need for laser surgery or intraocular injections of anti-vascular endothelial growth factor agents. NEC will be defined as ≥ stage 2 according to Bell’s criteria [40] and BPD as oxygen requirement at 36 weeks post-menstrual age [41].

Secondary outcomes to be compared among iNeo member networks will include the individual morbidities of the composite outcome, as well as nosocomial infection defined as culture-proven sepsis (blood or cerebrospinal fluid positive for pathogenic organism) at >3 days or 72 hours postnatal age [42], patent ductus arteriosus requiring pharmacological treatment and/or surgical ligation, receipt of delivery room cardiopulmonary resuscitation, air leak syndrome, and resource utilization (length of stay and length of respiratory support). To account for potential differences in practices regarding discharge home and transfer to Level 2 community units, additional analyses will compare mortality by Day 28 after birth. All outcomes will be expressed as ratios with the denominator equal to all admissions to participating NICUs.

#### Adjustment for variations in baseline population characteristics between networks

Demographic characteristics and severity of illness are well known to impact neonatal outcomes [43] and are also likely to vary between networks. To prevent bias, these potential confounders will be controlled in analyses comparing network-level outcome rates. The common minimum dataset includes important predictors, such as gestational age, sex, plurality of pregnancy, and receipt of antenatal corticosteroids, which will be used to adjust analyses as appropriate. In addition, most networks collect various measures of ‘severity of illness’, such as CRIB [44], SNAPPE-II [45], or TRIPS [46] scores. These will be standardized within each network (assigned a score between 0 and 1) and adjusted for in analyses.

#### Descriptive analyses of baseline factors

The distribution of infant characteristics and network-level broad organizational structural features will be summarized as counts and percentages for categorical variables and using the mean and standard deviation, or the median and interquartile range for continuous variables. The data will be compared among all networks using the Chi-square test for categorical and ANOVA F-test or Mood’s median test for continuous variables.

#### Comparisons between networks

For the primary composite outcome, each of its components and the additional secondary outcomes, initial crude rates, and associated 95% and 99% confidence intervals will be calculated and graphically displayed using ‘caterpillar plots’ to visually identify differences between networks. To adjust for multiple baseline characteristics, standardized outcome ratios will be computed using the ‘indirect standardization’ approach. Each network’s observed rate will be compared with the expected rate based on the total sample from all other networks to identify networks with rates significantly above or below average. For each outcome, the expected number of events will be computed as the sum of predicted probabilities from a multivariable model (logistic regression or zero inflated negative binomial models based on data distribution) derived using data from all other networks with adjustment for confounders. Network standardized outcome ratios will be graphically displayed using ‘funnel’ plots with 95% and 99% prediction intervals for comparison between networks.

A global comparison, as well as pair-wise comparisons between networks, will be performed using multivariate regression models adjusted for confounders. Statistical models will employ generalized estimating equations to adjust analyses for clustering of infants within networks. In addition, hierarchical random-effects regression models will be used to allow for variation at the network and unit level. Statistical significance will be evaluated by applying a Bonferroni correction to account for multiple pair-wise comparisons.

#### Statistical power for outcome comparisons

With retrospective data from 251 NICUs collected over four years (2007–2010), analyses (two-sided tests) comparing Canada (10,800 admissions) with all other networks (82,800 admissions), for example, will be able to detect rate differences of 0.004 to 0.02 for a range of outcome rates (1% to 40%) with statistical power of 80% assuming 5% type I error rate. Similar analyses comparing Canada with one other network (3,200 to 30,800 admissions) will be able to detect rate differences of 0.007 to 0.03.

### Association of site characteristics and practices with outcomes

To identify factors contributing to outcome variation between networks, detailed information will be obtained on health service provision, including units’ physical layout, environmental characteristics, human factors, and management practices at the national and site level. The type of data and strategy for collecting this information will be determined following the comparison of outcomes between networks to target identified problem areas and evaluate the culture, context, and practices of each network. Factors with possible impact on outcome differences between and within networks will be ascertained using a variety of tools, such as surveys, recurring questionnaires, and in specific instances, site visits to explore details if permitted.

The data will be pooled across sites and networks, and statistical analyses will identify factors significantly associated with outcomes. Through a collaborative process, findings will be discussed with members of participating networks to select physical and environmental factors, human and resource factors, or processes of care that can be modified through a quality improvement process. Each network will then implement practice changes within these three main target areas according to their outcome priorities and the constraints of their respective health care systems.

#### Physical and environmental factors

For preterm infants, adaptation to the environment is crucial for their survival, wellbeing, and development. The physical environment of the NICU is significantly different from the in-utero environment and contains a wide range of sensory stimuli that a preterm infant would not be exposed to if carried to term [47]. There has been wide debate as to the optimal physical characteristics of a NICU in relation to outcomes for VLBW infants. Several units that have implemented a single infant per room design in place of the more traditional open multi-patient rooms have reported improvements in outcomes, but impact on staff satisfaction and work-efficiency remains unclear [48,49]. Higher physical demands and workloads placed on nurses could negatively affect the level of care provided. Additional key physical characteristics include internal and external noise [50,51], temperature control, exposure to light [52,53], practice of developmentally supportive care [54], provision and extent of family-centered care, provision and extent of breastfeeding support, potential for continuous parental involvement, as well as training and preparation for discharge home.

Physical characteristics will be assessed by conducting a snapshot survey of units within the iNeo networks. The survey will be developed, piloted, and implemented in collaboration with the iNeo Scientific Advisory Committee by iNeo researchers with experience investigating the extraneous factors that may impact quality of care.

#### Human and resource factors

Human factors and available resources represent another aspect of care provision possibly associated with differences in outcomes. However, associations between human and resource factors and neonatal outcomes have not been thoroughly investigated, particularly not on a national scale. Human factors include staffing in relation to day and night shifts [33,55], weekdays versus weekends [56], ratio of nurses to patients [57], pattern of work for medical and nursing staff (hours on call, total duration of active duty time over 4 week period, etc.), number and types of trainee doctors, allied healthcare personnel coverage, constitution of attending team for high-risk births, and relative expertise of the health care providers attending resuscitation of extremely preterm infants considering their overall experience in direct patient care, training, and research.

Neonatal outcomes are also impacted by resource availability and utilization, specifically volume and capacity. Units with high volume are reported to have better outcomes compared with units with low volume, possibly due to relatively increased staff experience [58,59]; however, it has also been noted that low volume units may be less crowded and have reduced rates of complications [60]. Alternatively, these differences may be secondary to centralization of care rather than volume, as seen in data from Finland [61]. Similarly, units functioning at >90% capacity at all times, irrespective of volume, may have different outcomes compared with units operating at lower capacity.

Data on human factors and resource utilization will be collected using snapshot surveys administered at the unit level. Due to likely variations from year to year, data on human factors and resource utilization will be collected on an annual basis using electronic tools (such as recurring auto-filled surveys based on previous responses so as to only report changes), and while the data may not capture variation in the daily activity levels or acuity in the unit, this will represent the average condition.

#### Care-provision factors

Clinical practices represent the third and possibly most important set of characteristics that likely contribute to variation in outcomes. Variations in clinical practices are well known among neonatal communities [8]; however, no systematic prospective approach has determined, compared, and benchmarked variations associated with outcomes. Some of the key practice variations between centers and networks include referral practices (inborn vs. outborn) [62-64], differential use of the type of initial respiratory support [65-67], types and timings of surfactant administration [68], fluid management [69], timing of initiation of parenteral nutrition [70], use of donor milk, management of patent ductus arteriosus [71], availability and use of echocardiography, use of prophylactic interventions [72] (e.g., probiotics, high frequency oscillatory ventilation, phototherapy, and L-arginine), and the scope of involvement of parents.

Specific to each secondary outcome we will identify ‘top’ performing networks and networks with significant room for improvement. Subsequently, working groups of interested stakeholders from each network will be formed to determine methods to identify possible care provision practices related to such variations. Study methods will be similar to those described earlier, and will include annual snapshot surveys of each unit, detailed questionnaires specific to practices (e.g. parental presence, use of donor milk, diagnosis and management of hypotension, etc.), and in certain instances of outstanding success, a site visit with structured exploration of the practices in question. All methods of exploration will be conducted with directions from the iNeo Governing Board and Scientific Advisory Committee to protect privacy and confidentiality. Because individual unit information will not be disclosed to the iNeo Coordinating Centre, individual networks will be asked to identify willing members for such participation.

#### Statistical analyses and power for identification of practice and service variation

Associations of clinical management practices and other external factors with outcomes will be assessed under the general framework of individual patient-level data meta-analyses. Random-effects models with adjustment for confounding variables and important risk factors will provide estimates of association and quantify residual variation due to unknown or unmeasured unit-specific and network-level factors. These analyses will identify treatment practices and health care services with significant impact on outcomes, which subsequently can be targeted for implementation or improvement by specific units or networks. This information along with details of the practices/factors will be made available to initiate discussion within the iNeo community regarding data-informed, evidence-linked potentially better practices. Analyses (two-sided tests) based on 10,000 yearly admissions evaluating impact of treatment/practices (assuming 50% exposure) on outcomes (incidence 1% to 40%) will be able to detect relative risks of 1.6 to 1.1 with statistical power of 80% and 5% type I error rate. This is a conservative power calculation based on data expected to be collected in a one-year timeframe.

### Implementation and evaluation of practice changes to improve outcomes

Practices identified as being associated with an improvement in outcomes will be proposed to network sites for implementation using the continuous cycle of application and evaluation central to the EPIQ method [34,35]. Quality improvement using EPIQ methodology has been implemented in Canadian NICUs for the last 10 years. It is based on three pillars: (1) the use of all available evidence on a particular intervention from the published scientific literature, (2) analysis of each institute’s baseline data to identify hospital-specific practices for targeted intervention, and (3) the use of a network to share the results of quality improvement for the purpose of collaborative learning. The EPIQ method utilizes local context and allows customization of interventions and implementation strategies to maximize improvement potential at each institute. This is conducted in conjunction with leadership and peer support from network members [34,35].

Our plan for the iNeo network is to expand the EPIQ approach to an international level. We will advocate incorporation of several cycles of practice change implementation, evaluation, monitoring, and collaborative learning within each unit over the course of two and a half years. The online ViviWeb Virtual Research Community (https://meta.cche.net/viviweb/default.asp) will be used to facilitate collaboration between networks. Based on our experiences and preliminary results implementing practice changes in Canada, and following discussion with the NRNJ, we anticipate that regular and productive dialogue will significantly benefit many of the participating NICUs.

The practice changes implemented by individual units within networks will be evaluated every 6 to 12 months depending upon each centre’s capabilities to collect and submit data. In addition to outcome indicators, process indicators will be developed based on the specific interventions implemented. These indicators will measure the short-term impact of practice change. For example, an intervention targeting early surfactant administration to reduce BPD will have process indicators for the time of first surfactant administration and the proportion of babies who received surfactant within the first 30 minutes after birth. The outcome of interest for this intervention will be reduction in the incidence of BPD. Safety and outcome improvements will be monitored within each unit and network using control charts and Chi-square tests for differences in outcome rates from baseline. Multivariable logistic regression analyses will pool data from units within each network to assess changes in outcomes over time with adjustment for potential confounders and important risk factors, and accounting for clustering.

### Long-term neurodevelopmental follow-up

The members of iNeo have agreed that while the present initiative should focus on ascertaining outcomes prior to discharge from the NICU, the longer-term goal should be to assess and improve neurodevelopmental outcomes of VLBW and VPT infants at two to three years of age. Presently, five networks (CNN, NRNJ, NDAU, SNN, and ANZNN) follow and collect data from their infants up to two to three years of age with one more network in the planning stages of follow-up data collection (SNQ). The remaining networks have expressed interest in long-term follow-up, and will explore the possibility of collecting these data. For available follow-up data, extraneous factors, and process of care factors during NICU stay will be examined in relation to outcomes at two to three years of age. A composite severe adverse outcome will be defined as mortality or severe morbidity, including non-ambulatory cerebral palsy, developmental indices more than two standard deviations below the mean, legal blindness, or deafness requiring amplification. This will require development of a follow-up dataset (similar to the NICU minimum dataset) for the long-term neurodevelopmental outcomes.

### Secondary research questions

In order to foster a true international collaboration, the data collected and housed at the iNeo Coordinating Centre will be available to all iNeo member networks and iNeo-affiliated investigators after the principal analyses are completed. The iNeo database will be available to iNeo-affiliated investigators, including trainees, wishing to examine new research questions/hypotheses. Requests for data will need to be sent to the iNeo Coordinating Centre for discussion and approval by the iNeo Scientific Advisory Committee. In the initial stages of the iNeo collaboration, analysis of the dataset in question will be performed at the iNeo Coordinating Centre and the results sent to the requesting investigator. In the later stages, limited datasets may be released to an investigator using a secure electronic portal system. In all publications, the final author will be ‘the International Network for Evaluating Outcomes of Neonates (iNeo)’. For the analyses detailed in this protocol, the author list will include representatives of all eight networks. For additional projects, authors will be those individuals who meet the criteria for authorship as laid out by the ICJME. All publications will include a list of the member networks in the acknowledgements.

## Discussion

The iNeo collaboration will be the first multi-national network to examine population-based data. Findings from this international collaboration generated using extensive data will provide strong and novel evidence regarding practices contributing to outcome variation with broad relevance to NICUs within iNeo and worldwide. This is particularly true for the investigation of the environmental, human, and physical factors that impact neonatal outcomes. The majority of current literature relates to single center or regional experiences, whereas data from multiple national networks will provide robust estimates that will allow development of unified recommendations regarding optimal design and staffing of neonatal units.

The nature of the information that will be generated and the resources available within the collaborative will put iNeo in a unique position to implement global change to improve neonatal outcomes. Neonatal outcomes and NICU care practices will likely vary significantly between networks and there are many factors that may underlie these variations. The initial findings from the comparative analysis may not be welcomed by all units, and recommendations for practice changes that require extensive change or high financial input, such as additional staff to attend births or changes to unit layout, may be met with resistance. In answer to this, the most persuasive element of the iNeo collaboration will be the strength of the evidence produced from the data, the pragmatic nature of the results, and higher degree of statistical precision due to the large sample size.

In addition to the strength of the data, a high level of collaboration between network members will provide a mechanism to address barriers to change and ensure the knowledge gained is effectively implemented to improve neonatal outcomes. Working together we will ensure that all factors that contribute to a target outcome are identified and evaluated. Once identified, the process for exploration of extraneous factors will be supervised by the iNeo Director and Scientific Advisory Committee to ensure that all suggested practice changes can be tailored to networks depending on the presence or absence of certain baseline covariates. Although the individual network directors will be primarily responsible for driving change within their networks, iNeo will also provide various activities and mechanisms to facilitate practice change. This will include access to in-person and online training, site visits between networks, effective dissemination of information, and liaison with policy makers in member countries.

The iNeo collaboration will also act as a platform whereby other NICUs and established networks or networks in the preliminary phase of development can access evidence regarding impact of practices on outcomes, and approaches for collaborative learning and practice improvement in neonatology. As such, initial discussions with neonatal units in India, China, South America, and Taiwan have been productive and these networks are planning to assess and apply the results of the iNeo collaboration.

In summary, the iNeo collaboration will serve as a strong international platform for neonatal-perinatal health services research in VLBW and VPT infants. The evidence obtained using the iNeo platform will enable clinical teams from member networks to identify, implement, and evaluate practice and service provision changes aimed at improving the care and outcomes of VLBW and VPT infants within their respective countries. The knowledge generated, assembly of expertise, and pool of resources will be available worldwide with a likely global impact.

## Abbreviations

ANZNN: Australia-New Zealand Neonatal Network; BPD: Bronchopulmonary dysplasia; CNN: Canadian Neonatal Network; EPIQ: Evidence-based Practice for Quality Improvement; iNeo: International Network for Evaluating Outcomes of Neonates; INN: Israel Neonatal Network; IVH: Intraventricular hemorrhage; NEC: Necrotizing enterocolitis; NI: Nosocomial infection; NICU: Neonatal intensive care unit; NRNJ: Neonatal Research Network of Japan; SNN: Swiss Neonatal Network; SNQ: Swedish Neonatal Quality Register: Neonatology; ROP: Retinopathy of prematurity; SEN1500: Spanish Neonatal Network; UKNC: UK Neonatal Collaborative; VLBW: Very low birth weight; VPT: Very preterm.

## Competing interests

The authors declare that they have no competing interests.

## Authors’ contributions

PSS conceived of the concept of iNeo, led the protocol design process, and drafted the manuscript. LM designed the statistical analysis plan and participated in the protocol design process. All the remaining authors (SKL, KL, GS, RM, BR, SH, LSF, NM, MA, BD, MF, SK, RH) participated in network and protocol design including reaching consensus on the minimum dataset, and will direct the collection of data, dissemination of knowledge, and implementation of practice changes within their respective networks. All authors read, revised, and approved the final manuscript.

## Pre-publication history

The pre-publication history for this paper can be accessed here:

http://www.biomedcentral.com/1471-2431/14/110/prepub

## Supplementary Material

Additional file 1 **iNeo data variables for collection with explanatory notes.** Description: List of the data variables that will be collected and analyzed during the project described in the iNeo protocol. Click here for file

## References

[B1] Blencowe H Cousens S Oestergaard MZ Chou D Moller AB Narwal R Adler A Vera GC Rohde S Say L Lawn JE National, regional, and worldwide estimates of preterm birth rates in the year 2010 with time trends since 1990 for selected countries: a systematic analysis and implications Lancet 2012 379 2162 2172 10.1016/S0140-6736(12)60820-4 22682464

[B2] Joseph KS Kramer MS Marcoux S Ohlsson A Wen SW Allen A Platt R Determinants of preterm birth rates in Canada from 1981 through 1983 and from 1992 through 1994 N Engl J Med 1998 339 1434 1439 10.1056/NEJM199811123392004 9811918

[B3] Statistics Canada Births 2012 84F0210X

[B4] Kliegman RM Rottman CJ Behrman RE Strategies for the prevention of low birth weight Am J Obstet Gynecol 1990 162 1073 1083 10.1016/0002-9378(90)91320-C 2183614

[B5] McCormick MC The contribution of low birth weight to infant mortality and childhood morbidity N Engl J Med 1985 312 82 90 10.1056/NEJM198501103120204 3880598

[B6] Moutquin J Milot-Roy VI Preterm birth prevention: effectiveness of current strategies J Soc Obstet Gynecol Can 1996 18 571 588

[B7] Cust AE Darlow BA Donoghue DA Outcomes for high risk New Zealand newborn infants in 1998–1999: a population based, national study Arch Dis Child Fetal Neonatal Ed 2003 88 F15 F22 10.1136/fn.88.1.F15 12496221 PMC1756015

[B8] Lee SK McMillan DD Ohlsson A Pendray M Synnes A Whyte R Chien LY Sale J Variations in practice and outcomes in the Canadian NICU network: 1996–1997 Pediatrics 2000 106 1070 1079 10.1542/peds.106.5.1070 11061777

[B9] Bader D Kugelman A Boyko V Levitzki O Lerner-Geva L Riskin A Reichman B Risk factors and estimation tool for death among extremely premature infants: a national study Pediatrics 2010 125 696 703 10.1542/peds.2009-1607 20351002

[B10] Kusuda S Fujimura M Sakuma I Aotani H Kabe K Itani Y Ichiba H Matsunami K Nishida H Morbidity and mortality of infants with very low birth weight in Japan: center variation Pediatrics 2006 118 e1130 e1138 10.1542/peds.2005-2724 16950943

[B11] Fellman V Hellstrom-Westas L Norman M Westgren M Kallen K Lagercrantz H Marsal K Serenius F Wennergren M One-year survival of extremely preterm infants after active perinatal care in Sweden JAMA 2009 301 2225 2233 19491184 10.1001/jama.2009.771

[B12] Gale C Santhakumaran S Nagarajan S Statnikov Y Modi N Impact of managed clinical networks on NHS specialist neonatal services in England: population based study BMJ 2012 344 e2105 10.1136/bmj.e2105 22490978 PMC3318112

[B13] Fanaroff AA Hack M Walsh MC The NICHD neonatal research network: changes in practice and outcomes during the first 15 years Semin Perinatol 2003 27 281 287 10.1016/S0146-0005(03)00055-7 14510318

[B14] Horwood SP Boyle MH Torrance GW Sinclair JC Mortality and morbidity of 500- to 1,499-gram birth weight infants live-born to residents of a defined geographic region before and after neonatal intensive care Pediatrics 1982 69 613 620 7079020

[B15] Lemons JA Bauer CR Oh W Korones SB Papile LA Stoll BJ Verter J Temprosa M Wright LL Ehrenkranz RA Fanaroff AA Stark A Carlo W Tyson JE Donovan EF Shankaran S Stevenson DK Very low birth weight outcomes of the National Institute of Child health and human development neonatal research network, January 1995 through December 1996. NICHD Neonatal Research Network Pediatrics 2001 107 E1 10.1542/peds.107.1.e1 11134465

[B16] Hack M Wright LL Shankaran S Tyson JE Horbar JD Bauer CR Younes N Very-low-birth-weight outcomes of the National Institute of Child Health and Human Development Neonatal Network, November 1989 to October 1990 Am J Obstet Gynecol 1995 172 457 464 10.1016/0002-9378(95)90557-X 7856670

[B17] Horbar JD Badger GJ Carpenter JH Fanaroff AA Kilpatrick S LaCorte M Phibbs R Soll RF Trends in mortality and morbidity for very low birth weight infants, 1991–1999 Pediatrics 2002 110 143 151 10.1542/peds.110.1.143 12093960

[B18] Meadow W Lee G Lin K Lantos J Changes in mortality for extremely low birth weight infants in the 1990s: implications for treatment decisions and resource use Pediatrics 2004 113 1223 1229 10.1542/peds.113.5.1223 15121933

[B19] Shah PS Sankaran K Aziz K Allen AC Seshia M Ohlsson A Lee SK Outcomes of preterm infants <29 weeks gestation over 10-year period in Canada: a cause for concern? J Perinatol 2012 32 132 138 10.1038/jp.2011.68 21593814

[B20] Draper ES Zeitlin J Field DJ Manktelow BN Truffert P Mortality patterns among very preterm babies: a comparative analysis of two European regions in France and England Arch Dis Child Fetal Neonatal Ed 2007 92 F356 F360 10.1136/adc.2006.097683 17213271 PMC2675356

[B21] Draper ES Zeitlin J Fenton AC Weber T Gerrits J Martens G Misselwitz B Breart G Investigating the variations in survival rates for very preterm infants in 10 European regions: the MOSAIC birth cohort Arch Dis Child Fetal Neonatal Ed 2009 94 F158 F163 18805823 10.1136/adc.2008.141531

[B22] Evans N Hutchinson J Simpson JM Donoghue D Darlow B Henderson-Smart D Prenatal predictors of mortality in very preterm infants cared for in the Australian and New Zealand Neonatal Network Arch Dis Child Fetal Neonatal Ed 2007 92 F34 F40 10.1136/adc.2006.094169 16877475 PMC2675296

[B23] Field D Bajuk B Manktelow BN Vincent T Dorling J Tarnow-Mordi W Draper ES Smart DH Geographically based investigation of the influence of very-preterm births on routine mortality statistics from the UK and Australia Arch Dis Child Fetal Neonatal Ed 2008 93 F212 F216 10.1136/adc.2007.119271 17916593

[B24] Parry GJ Gould CR McCabe CJ Tarnow-Mordi WO Annual league tables of mortality in neonatal intensive care units: longitudinal study. International Neonatal Network and the Scottish Neonatal Consultants and Nurses Collaborative Study Group BMJ 1998 316 1931 1935 10.1136/bmj.316.7149.1931 9641927 PMC28588

[B25] Richardus JH Graafmans WC Verloove-Vanhorick SP Mackenbach JP The perinatal mortality rate as an indicator of quality of care in international comparisons Med Care 1998 36 54 66 10.1097/00005650-199801000-00007 9431331

[B26] Ishii N Kono Y Yonemoto N Kusuda S Fujimura M Outcomes of infants born at 22 and 23 weeks’ gestation Pediatrics 2013 132 62 71 10.1542/peds.2012-2857 23733804

[B27] UK Chief Medical Officer Atlas of Variation in Healthcare for Children and Young People, Annex of the Annual Report of the Chief Medical Officer 2012 2013 Our Children Deserve Better: Prevention Pays

[B28] Risk adjusted and population based studies of the outcome for high risk infants in Scotland and Australia International Neonatal Network, Scottish Neonatal Consultants, Nurses Collaborative Study Group Arch Dis Child Fetal Neonatal Ed 2000 82 F118 F123 10.1136/fn.82.2.F118 10685984 PMC1721047

[B29] Sexson WR Cruze DK Escobedo MB Brann AW Report of an international conference on the medical and ethical management of the neonate at the edge of viability: a review of approaches from five countries HEC Forum 2011 23 31 42 10.1007/s10730-011-9149-6 21424778

[B30] Isayama T Lee SK Mori R Kusuda S Fujimura M Ye XY Shah PS Comparison of Mortality and Morbidity of Very Low Birth Weight Infants Between Canada and Japan Pediatrics 2012 130 e957 e965 10.1542/peds.2012-0336 22966031

[B31] Hossain S Shah PS Lee SK Darlow B Sullivan E Lui K Comparison of characteristics and outcomes of very preterm infants admitted to Australia-New Zealand and Canadian neonatal networks [abstract] Pediatr Acad Soc 2012

[B32] Shah PS Hossain S Lui K Darlow B Gallimore V Lee SK Outcomes of outborn very preterm infants in Australia-New Zealand and in Canada [abstract] Pediatr Acad Soc 2012

[B33] Abdel-Latif ME Bajuk B Oei J Lui K Mortality and morbidities among very premature infants admitted after hours in an Australian neonatal intensive care unit network Pediatrics 2006 117 1632 1639 10.1542/peds.2005-1421 16651317

[B34] Lee SK Aziz K Singhal N Cronin CM James A Lee DS Matthew D Ohlsson A Sankaran K Seshia M Synnes A Walker R Whyte R Langley J Macnab YC Stevens B von Dadelszen P Improving the quality of care for infants: a cluster randomized controlled trial CMAJ 2009 181 469 476 10.1503/cmaj.081727 19667033 PMC2761437

[B35] Cronin CM Baker GR Lee SK Ohlsson A McMillan DD Seshia MM Reflections on knowledge translation in Canadian NICUs using the EPIQ method Healthc Q 2011 3 8 16 14 Spec No 22008567 10.12927/hcq.2011.22539

[B36] World Health Organization International Statistical Classification of Diseases and Related Health Problems 10th Revision (ICD-10) 2010 http://apps.who.int/classifications/icd10/browse/2010/en

[B37] International Health Terminology Standards Development Organisation. SNOMED CT 2012 http://www.ihtsdo.org/snomed-ct/

[B38] Papile LA Burstein J Burstein R Koffler H Incidence and evolution of subependymal and intraventricular hemorrhage: a study of infants with birth weights less than 1,500 gm J Pediatr 1978 92 529 534 10.1016/S0022-3476(78)80282-0 305471

[B39] International Committee for the Classification of Retinopathy of Prematurity The International Classification of Retinopathy of Prematurity revisited Arch Ophthalmol 2005 123 991 999 16009843 10.1001/archopht.123.7.991

[B40] Bell MJ Ternberg JL Feigin RD Keating JP Marshall R Barton L Brotherton T Neonatal necrotizing enterocolitis. Therapeutic decisions based upon clinical staging Ann Surg 1978 187 1 7 10.1097/00000658-197801000-00001 413500 PMC1396409

[B41] Shennan AT Dunn MS Ohlsson A Lennox K Hoskins EM Abnormal pulmonary outcomes in premature infants: prediction from oxygen requirement in the neonatal period Pediatrics 1988 82 527 532 3174313

[B42] Freeman J Epstein MF Smith NE Platt R Sidebottom DG Goldmann DA Extra hospital stay and antibiotic usage with nosocomial coagulase-negative staphylococcal bacteremia in two neonatal intensive care unit populations Am J Dis Child 1990 144 324 329 2305739 10.1001/archpedi.1990.02150270074029

[B43] Shah PS Ye XY Synnes A Rouvinez-Bouali N Yee W Lee SK Prediction of survival without morbidity for infants born at under 33 weeks gestational age: a user-friendly graphical tool Arch Dis Child Fetal Neonatal Ed 2012 97 F110 F115 10.1136/archdischild-2011-300143 21900280

[B44] Parry G Tucker J Tarnow-Mordi W CRIB II: an update of the clinical risk index for babies score Lancet 2003 361 1789 1791 10.1016/S0140-6736(03)13397-1 12781540

[B45] Richardson DK Corcoran JD Escobar GJ Lee SK SNAP-II and SNAPPE-II: Simplified newborn illness severity and mortality risk scores J Pediatr 2001 138 92 100 10.1067/mpd.2001.109608 11148519

[B46] Lee SK Zupancic JA Pendray M Thiessen P Schmidt B Whyte R Shorten D Stewart S Transport risk index of physiologic stability: a practical system for assessing infant transport care J Pediatr 2001 139 220 226 10.1067/mpd.2001.115576 11487747

[B47] Kuhn P Zores C Astruc D Dufour A Casper C [Sensory system development and the physical environment of infants born very preterm] Arch Pediatr 2011 18 Suppl 2 S92 S102 21763981 10.1016/S0929-693X(11)71097-1

[B48] Domanico R Davis DK Coleman F Davis BO Documenting the NICU design dilemma: comparative patient progress in open-ward and single family room units J Perinatol 2011 31 281 288 10.1038/jp.2010.120 21072040 PMC3070087

[B49] Domanico R Davis DK Coleman F Davis BO Jr Documenting the NICU design dilemma: parent and staff perceptions of open ward versus single family room units J Perinatol 2010 30 343 351 10.1038/jp.2009.195 20072132 PMC2864417

[B50] Darcy AE Hancock LE Ware EJ A descriptive study of noise in the neonatal intensive care unit. Ambient levels and perceptions of contributing factors Adv Neonatal Care 2008 8 165 175 10.1097/01.ANC.0000324341.24841.6e 18535422

[B51] Liu WF Comparing sound measurements in the single-family room with open-unit design neonatal intensive care unit: the impact of equipment noise J Perinatol 2012 32 368 373 10.1038/jp.2011.103 21852773

[B52] Lasky RE Williams AL Noise and light exposures for extremely low birth weight newborns during their stay in the neonatal intensive care unit Pediatrics 2009 123 540 546 10.1542/peds.2007-3418 19171620

[B53] Morag I Ohlsson A Cycled light in the intensive care unit for preterm and low birth weight infants Cochrane Database Syst Rev 2011 CD006982 10.1002/14651858.CD006982.pub2 21249685

[B54] Als H NIDCAP: testing the effectiveness of a relationship-based comprehensive intervention Pediatrics 2009 124 1208 1210 10.1542/peds.2009-1646 19786455

[B55] Lee SK Lee DS Andrews WL Baboolal R Pendray M Stewart S Higher mortality rates among inborn infants admitted to neonatal intensive care units at night J Pediatr 2003 143 592 597 10.1067/S0022-3476(03)00367-6 14615728

[B56] Salihu HM Ibrahimou B August EM Dagne G Risk of infant mortality with weekend versus weekday births: A population-based study J Obstet Gynaecol Res 2012 10.1111/j.1447-0756.2011.01818.x 22487462

[B57] Mefford LC Alligood MR Evaluating nurse staffing patterns and neonatal intensive care unit outcomes using Levine’s Conservation Model of Nursing J Nurs Manag 2011 19 998 1011 10.1111/j.1365-2834.2011.01319.x 22074302

[B58] Grandi C Gonzalez A Meritano J [Patient volume, medical and nursing staffing and its relationship with risk-adjusted outcomes of VLBW infants in 15 Neocosur neonatal network NICUs] Arch Argent Pediatr 2010 108 499 510 21132246 10.1590/S0325-00752010000600005

[B59] Phibbs CS Bronstein JM Buxton E Phibbs RH The effects of patient volume and level of care at the hospital of birth on neonatal mortality JAMA 1996 276 1054 1059 10.1001/jama.1996.03540130052029 8847767

[B60] Tucker J Patient volume, staffing, and workload in relation to risk-adjusted outcomes in a random stratified sample of UK neonatal intensive care units: a prospective evaluation Lancet 2002 359 99 107 11809250 10.1016/s0140-6736(02)07366-x

[B61] Rautava L Lehtonen L Peltola M Korvenranta E Korvenranta H Linna M Hallman M Andersson S Gissler M Leipala J Tammela O Hakkinen U PERFECT Preterm Infant Study Group: The effect of birth in secondary- or tertiary-level hospitals in Finland on mortality in very preterm infants: a birth-register study Pediatrics 2007 119 e257 e263 10.1542/peds.2006-1964 17200251

[B62] Chien LY Whyte R Aziz K Thiessen P Matthew D Lee SK Improved outcome of preterm infants when delivered in tertiary care centers Obstet Gynecol 2001 98 247 252 10.1016/S0029-7844(01)01438-7 11506840

[B63] Lui K Abdel-Latif ME Allgood CL Bajuk B Oei J Berry A Henderson-Smart D Improved outcomes of extremely premature outborn infants: effects of strategic changes in perinatal and retrieval services Pediatrics 2006 118 2076 2083 10.1542/peds.2006-1540 17079581

[B64] Shah PS Shah V Qiu Z Ohlsson A Lee SK Improved outcomes of outborn preterm infants if admitted to perinatal centers versus freestanding pediatric hospitals J Pediatr 2005 146 626 631 10.1016/j.jpeds.2005.01.030 15870665

[B65] Vanpee M Walfridsson-Schultz U Katz-Salamon M Zupancic JA Pursley D Jonsson B Resuscitation and ventilation strategies for extremely preterm infants: a comparison study between two neonatal centers in Boston and Stockholm Acta Paediatr 2007 96 10 16 10.1111/j.1651-2227.2007.00063.x 17187596

[B66] Tingay DG Mills JF Morley CJ Pellicano A Dargaville PA Trends in use and outcome of newborn infants treated with high frequency ventilation in Australia and New Zealand, 1996–2003 J Paediatr Child Health 2007 43 160 166 10.1111/j.1440-1754.2007.01036.x 17316190

[B67] Roberts CL Badgery-Parker T Algert CS Bowen JR Nassar N Trends in use of neonatal CPAP: a population-based study BMC Pediatr 2011 11 89 10.1186/1471-2431-11-89 21999325 PMC3206424

[B68] van Kaam AH De Jaegere AP Borensztajn D Rimensberger PC Surfactant replacement therapy in preterm infants: a European survey Neonatology 2011 100 71 77 10.1159/000322004 21228602

[B69] Oh W Poindexter BB Perritt R Lemons JA Bauer CR Ehrenkranz RA Stoll BJ Poole K Wright LL Association between fluid intake and weight loss during the first ten days of life and risk of bronchopulmonary dysplasia in extremely low birth weight infants J Pediatr 2005 147 786 790 10.1016/j.jpeds.2005.06.039 16356432

[B70] Kotsopoulos K Benadiba-Torch A Cuddy A Shah PS Safety and efficacy of early amino acids in preterm <28 weeks gestation: prospective observational comparison J Perinatol 2006 26 749 754 10.1038/sj.jp.7211611 17024139

[B71] Lai LS McCrindle BW Variation in the diagnosis and management of patent ductus arteriosus in premature infants Paediatr Child Health 1998 3 405 410 20401223 10.1093/pch/3.6.405 PMC2851305

[B72] Clerihew L McGuire W Antifungal prophylaxis for very low birthweight infants: UK national survey Arch Dis Child Fetal Neonatal Ed 2008 93 F238 F239 10.1136/adc.2007.121830 17768153

