# Supplementary material for: The International Network for Evaluating Outcomes of very low birth weight, very preterm neonates (iNeo): a protocol for collaborative comparisons of international health services for quality improvement in neonatal care
Source: BMC Pediatr. 2014 Apr 23;14:110. doi: 10.1186/1471-2431-14-110 (PMC4021416; doi:10.1186/1471-2431-14-110)
Supplement: Additional file 1 — iNeo data variables for collection with explanatory notes. Description: List of the data variables that will be collected and analyzed during the project described in the iNeo protocol. [file 1471-2431-14-110-S1.pdf]

The International Network for Evaluating Outcomes of very low birth weight, very preterm neonates (iNeo): A protocol for collaborative comparisons of international health services for quality improvement in neonatal care

Prakesh S. Shah, Shoo K. Lee, Kei Lui, Gunnar Sjörs, Rintaro Mori, Brian Reichman, Stellan Håkansson, Laura San Feliciano, Neena Modi, Mark Adams, Brian Darlow, Masanori Fujimura, Satoshi Kusuda, Ross Haslam, Lucia Mirea, on behalf of the International Network for Evaluating Outcomes of Neonates (iNeo)

Additional file 1: iNeo data variables for collection with explanatory notes

iNeo data variables for collection with explanatory notes
Developed collaboratively by all participating countries

**ID**	Unique ID assigned by you or we will assign at coordinating center	
**Country**	Code for country (alphabetical list)	**Potential values:** 01 = Australia/New Zealand, 02 = Canada, 03 = Israel, 04 = Japan, 05 = Sweden, 06 = Switzerland, 07 = Spain, 08 = UK
**Center**	Code number only to be sent to iNeo	You need to prepare a list of centers and keep this code with you at your coordination center. In future surveys we will send link with center as code number and then we can link some of physical, organizational, environmental factors to the outcomes. The center assignment will be determined by the network but as a guide we suggest that the center that provided highest level of care (level 3 care) be assigned to that baby. If a baby has been admitted to two tertiary level units before discharge home then the first tertiary level NICU will be assigned to the baby.<br>**Potential values:** The center number will lie between 001 and 999
**Demographics**		
**Gestational age**	Weeks	**Potential values:** Range from 22 to 44
**Gestational age**	Days	**Potential values:** Range from 0 to 6
**Birth weight (kg)**		For units that can provide exact birth weight we would prefer this information. For units that have limitations or concerns regarding privacy, please provide BW info in 100 g intervals starting at 200-299, 300-399…….. etc.<br>**Potential values:**<br>**a.** Direct weight

		**b.** Range of weight<br>1 = 200-299<br>2 = 300-399<br>3 = 400-499<br>4 = 500-599<br>5 = 600-699<br>6 = 700-799<br>7 = 800-899<br>8 = 900-999<br>9 = 1000-1099<br>10 =1100-1199<br>11 = 1200-1299<br>12 = 1300-1399<br>13 = 1400-1499<br>14 = 1500-1599<br>15 = 1600-1699<br>16 = 1700-1799<br>17 =1800-1899<br>18 = 1900-1999<br>19 = >2000
**Birth year info**	Year	Data will be entered as 2007 onwards
**Birth quarter info**	Quarter	**Potential values:**<br>1 = January–March<br>2 = April–June<br>3 = July–Sept<br>4 = Oct–Dec
**Age at NICU admission**		**Potential values:** hh:mm<br>If admitted at birth then enter 00.00<br>**Unknown or missing – leave blank**

iNeo data variables for collection with explanatory notes
Developed collaboratively by all participating countries

**Sex**		**Potential values:**<br>1 = Male<br>2 = Female<br>3 = Ambiguous<br>4 = Unknown/missing
**Maternal age**		In 5 year groups starting at 11 years.<br>**Potential values:**<br>1 = 11–15<br>2 = 16–20<br>3 = 21–25<br>4 = 26–30<br>5 = 31–35<br>6 = 36–40<br>7 = 41–45<br>8 = 46–50<br>9 = 51–55<br>10 = 56–60<br>11 = >61<br>12 = Unknown
**Antenatal details**		
**Antenatal Corticosteroid use**		**Potential values:**<br>1 = Yes<br>2 = No<br>3 = Unknown/missing
**If YES, was it Complete/Incomplete**		**Potential values:**<br>1 = Complete (at least 2 doses that are 12 hours apart and 12 hours passed since last dose)<br>2 = Incomplete<br>3 = Unknown/missing
**ROM Duration**		**Potential values:**<br>1 = <24 hours (includes zero hours or rupture at the time

		of birth)<br>2 = 24 hours to 1 week<br>3 = >1 weeks<br>4 = Unknown/missing
**Maternal High blood pressure**	Includes all hypertensive disorders (pregnancy-induced and otherwise)	**Potential values:**<br>1 = Yes<br>2 = No<br>3 = Unknown/missing
**Maternal diabetes**	Includes all glucose homeostasis disorders (pregnancy-induced and otherwise)	**Potential values:**<br>1 = Yes<br>2 = No<br>3 = Unknown /missing
**Birth details**		
**Mode of delivery**		**Potential values:**<br>1 = Vaginal<br>2 = Caesarean section<br>3 = Unknown/missing
**Presentation**	At birth or closest to the time of birth	**Potential values:**<br>1 = Vertex<br>2 = Breech<br>3 = Other<br>4 = Unknown/missing
**Births this pregnancy**	If one fetus dies in-utero, code pregnancy as twin/triplet as appropriate and not singleton or twin based on live births	**Potential values:**<br>1 = Singleton<br>2 = Twin<br>3 = Triplet<br>4 = Higher order
**Birth order**		**Potential values:**<br>1 = First<br>2 = Second<br>3 = Third

		4 = Forth<br>5 = Unknown/missing
**Place of birth**	Inborn applies to tertiary care hospital where NICU is located. Outborn includes those babies who needed to be transferred to NICU after birth for ongoing neonatal care from facility other than hospital in which they are cared for	**Potential values:**<br>1 = Inborn<br>2 = Outborn<br>3 = Unknown/missing.
**Immediate postnatal care**		
**Need for intubation**	Within first 30 minutes of birth	**Potential values:**<br>1 = Yes<br>2 = No<br>3 = Unknown/missing
**Need for CPAP**	Within first 30 minutes of birth	**Potential values:**<br>1 = Yes<br>2 = No<br>3 = Unknown/missing
**Need for CPR during initial stabilization**	CPR includes chest compression and/or epinephrine within first 30 minutes of birth	**Potential values:**<br>1 = Yes<br>2 = No<br>3 = Unknown/missing
**Apgar at 1 minute**		**Potential values:**<br>0–10,<br>Unknown/missing = Leave blank
**Apgar at 5 minutes**		**Potential values:**<br>0–10<br>Unknown/missing = Leave blank
**Admission status**		

**Re-admission**		**Potential values:**<br>1 = Yes<br>2 = No<br>3 = Unknown/missing
**Admission Head Circumference (cm)**	Up to one decimal point	**Potential values:**<br>15.0 cm to 99.9 cm<br>Unknown/missing = 0.0
**Admission length (CM)**	Up to one decimal point	**Potential values:**<br>15.0 cm to 99.9 cm<br>Unknown/missing = 0.0
**Admission temperature (^{o}C)**	Up to one decimal point	**Potential values:**<br>20.0^{0} C to 45.0^{0} C<br>Unknown/missing = 0.0
**Severity of illness score used**	Whatever score is used by network	SNAP, SNAPII, SNAPPE, CRIB, CRIB II, TRIPS<br>Data will be collected on all scores.<br>Analyses will be performed based on standardized scores to compare severity of illness on admission scores as adjusted data<br>Please enter score in your range
**Neonatal course**		
**Duration of Supplemental O_2**	Days or hours (use midnight as cut-off)	Number range from 0–10000 (depending upon unit: hours or days)<br>If not applicable as infant did not receive it then put zero<br>If missing/unknown then leave blank
**Duration of CPAP**	Days or hours (use midnight as cut off)	Number range from 0–10000 (depending upon unit: hours or days)<br>If not applicable as infant did not receive then put zero<br>If missing/unknown then leave blank
**Duration of mechanical ventilation**	Days or hours (use midnight as cut off)	Number range from 0–10000 (depending upon unit: hours or days)<br>If not applicable as infant did not receive it then zero

		If missing/unknown then leave it blank
**Duration of TPN in days**	Days/hours (use midnight as cut off)	Number range from 0–10000 (depending upon unit: hours or days)<br>If not applicable as infant did not receive then put zero<br>If missing/unknown then leave blank
**Surfactant**	Yes/no	**Potential values:**<br>1 = Yes<br>2 = No<br>3 = Unknown/missing
**Diagnoses/Procedures**		
**PDA**	Clinical or echocardiographic diagnosis. We will collect information on method most commonly used in separate survey.	**Potential values:**<br>1 = Yes<br>2 = No<br>3 = Unknown /missing
**Indomethacin or other NSAID for PDA treatment**		**Potential values:**<br>1 = Yes<br>2 = No<br>3 = Unknown /missing
**Surgical ligation**		**Potential values:**<br>1 = Yes<br>2 = No<br>3 = Unknown /missing
**RDS**	Clinical and/or radiological criteria	**Potential values:**<br>1 = Yes<br>2 = No<br>3 = Unknown /missing
**Air leak**	Includes Pneumothorax, Pneumomediastinum	**Potential values:**<br>1 = Yes<br>2 = No

		3 = Unknown /missing
**Air leak requiring drainage**	Needle paracentesis or chest tube to be included	**Potential values:**<br>1 = Yes<br>2 = No<br>3 = Unknown/missing
**BPD at 28 days (need for supplemental oxygen)**	X-ray is not mandatory	**Potential values:**<br>1 = Yes<br>2 = No<br>3 = Unknown/missing
**BPD at 36 weeks (need for supplemental oxygen)**	X-ray is not mandatory	**Potential values:**<br>1 = Yes<br>2 = No<br>3 = Unknown/missing
**Postnatal steroid use for BPD**	Only systemic use (Not inhaled steroid use)	**Potential values:**<br>1 = Yes<br>2 = No<br>3 = Unknown/missing
**NEC (diagnosed using Bell's criteria)**	Stage 2 or higher only	**Potential values:**<br>1 = Yes<br>2 = No<br>3 = Unknown/missing
**Surgery for NEC (laparotomy/drainage)**		**Potential values:**<br>1 = Yes<br>2 = No<br>3 = Unknown/missing
**IVH (most severe grade) prior to discharge**	Classification could be SEH/GMH, IVH, VE, IPE	**Potential values:**<br>0 = No hemorrhage<br>1 = Grade 1 hemorrhage or subependymal hemorrhage or subependymal cucst or germinal matrix hemorrhage<br>2 = Intraventricular hemorrhage without ventricular

		dilatation<br>3 = Ventricular dilatation with intraventricular hemorrhage<br>4 = Intraparenchymal echogenicity suggestive of hemorrhage or infarction with or without IVH<br>5 = Unknown/missing<br>6 = No exam
**PVL or persistent parenchymal opacity**	Persistent intraparenchymal echolucency or cysts identified on US or MRI	**Potential values:**<br>1 = Yes<br>2 = No<br>3 = Unknown/missing<br>4 = No exam
**International ROP criteria**	In either eye, record the worst stage	**Potential values:**<br>0 = No ROP<br>1 = Stage 1 ROP<br>2 = Stage 2 ROP<br>3 = Stage 3 ROP<br>4 = Stage 4 ROP<br>5 = Stage 5 ROP<br>6 = Unknown/missing<br>7 = No eye exam done
**Treatment for ROP**	Anti-VEGF or laser	**Potential values:**<br>1 = Yes<br>2 = No<br>3 = Unknown/missing
**Early onset sepsis**	Infection within 72 hours (3 days) of birth – positive blood and/or CSF for pathogenic organism	**Potential values:**<br>1 = Yes<br>2 = No<br>3 = Unknown/missing
**Early onset sepsis**	If yes – organism	**Potential values:**<br>1 = E Coli<br>2 = GBS

		3 = Other Gram negative bacteria<br>4 = Other Gram positive bacteria<br>5 = Fungal<br>6 = Viral<br>7 = Other<br>8 = Unknown
**Late onset sepsis**	Infection after 3 days (or 72 hours of age) – positive blood and/or CSF for pathogenic organism	**Potential values:**<br>1 = Yes<br>2 = No<br>3 = Unknown/missing/ Not applicable in case of death prior to 3 days
**LOS episode 1**	Organism<br>If multiple organisms are grown in culture consider most virulent: Virulence should be counted as: Fungal – Gram negative (including E coli), Gram positive (including GBS), viral, mycoplasma, other, in that sequence.	**Potential values:**<br>1 = E Coli<br>2 = GBS<br>3 = Other Gram negative bacteria<br>4 = Other Gram positive bacteria<br>5 = Fungal<br>6 = Viral<br>7 = Other<br>8 = Unknown/missing
**LOS episode 1**	Postnatal age in days	Value range from 3 to 500<br>Leave this blank if only one episode of sepsis
**LOS episode 2**	Organism<br>If multiple organisms are grown in culture consider most virulent: Virulence should be counted as: Fungal – Gram negative (including E coli), Gram positive (including GBS), viral, mycoplasma, other, in that sequence.	**Potential values:**<br>1 = E Coli<br>2 = GBS<br>3 = Other Gram negative bacteria<br>4 = Other Gram positive bacteria<br>5 = Fungal<br>6 = Viral<br>7 = Other<br>8 = Unknown/missing

**LOS episode 2**	Postnatal age in days	Value range from 3 to 500<br>Leave this blank if only one episode of sepsis
**LOS episode 3**	Organism<br>If multiple organisms are grown in culture consider most virulent: Virulence should be counted as: Fungal – Gram negative (including E coli), Gram positive (including GBS), viral, mycoplasma, other, in that sequence.	**Potential values:**<br>1 = E Coli<br>2 = GBS<br>3 = Other Gram negative bacteria<br>4 = Other Gram positive bacteria<br>5 = Fungal<br>6 = Viral<br>7 = Other<br>8 = Unknown/missing
**LOS episode 3**	Postnatal age in days	Value range from 3 to 500
**LOS episode 4**	Postnatal age in days	Value range from 3 to 500
**LOS episode 5**	Postnatal age in days	Value range from 3 to 500
**Congenital anomalies**	Major congenital anomaly – defined as those life-threatening or likely to affect quality of way to a significant degree	**Potential values:**<br>1 = Yes<br>2 = No<br>3 = Unknown/missing
**Specified list to be developed based on ICD-10**	If yes to above	**Please mention all congenital anomalies you have for a particular infant. If they are coded, please give code number (ICD code). Centrally we will separate them in major and minor groups**
**Discharge Information**		
**Age at Discharge/death**	Postnatal age in calendar days	Number varies from 1 to 1000<br>If died before 24 hours – enter duration in hours and add “h” at the end
**Delivery Room Death**		**Potential values:**<br>1 = Yes<br>2 = No
**Death**	Prior to discharge from NICU	**Potential values:**<br>1 = Yes

		2 = No
**Cause of death if available**		Again any free text would be acceptable for this item and we will group them centrally.
**Discharge destination**		**Potential values:**<br>1 = Home<br>2 = Level 2 or HDU<br>3 = SCBU<br>4 = Pediatric ward<br>5 = PICU<br>6 = Out of area/country<br>7 = Rehabilitation/palliative care<br>8 = Unknown/missing<br>9 = Other<br>10 = Unknown
**Breastfeeding/breast milk at discharge**		**Potential values:**<br>1 = Yes<br>2 = No<br>3 = Unknown/missing
**Oxygen at discharge**		**Potential values:**<br>1 = Yes<br>2 = No<br>3 = Unknown/missing
**Weight at discharge/death**		In Kg, range 0.000 to 20.000<br>Leave blank if unknown (especially in case of death)
